# A culturally-specific education strategy to improve stroke health literacy in Vietnamese communities in South Western Sydney

**DOI:** 10.1016/j.dialog.2025.100211

**Published:** 2025-03-25

**Authors:** Jessica Ly, Christopher Blair, Helen Badge, Michael Camit, Khoi Do, Timmy Pham, Nicola Chappelow, Dennis J. Cordato, Mark W. Parsons

**Affiliations:** a Department of Neurology and Neurophysiology, Liverpool Hospital, Liverpool, NSW, Australia; b South Western Sydney Clinical School, University of New South Wales, Liverpool, NSW, Australia; c Ingham Institute for Applied Medical Research, Liverpool, NSW, Australia; d Health Literacy SWSLHD Multicultural Services, Liverpool Hospital, NSW, Australia

**Keywords:** Stroke, Health literacy, Cultural competency, Vietnamese people, Educational intervention, New South Wales, South Western Sydney

## Abstract

**Background:**

South-Western Sydney (SWS) is home to a large Vietnamese community, who are at higher risk of stroke and adverse health outcomes than Australian-born individuals. There is limited research on the effect of educational interventions on stroke literacy in Vietnamese communities. This study aimed to characterise recognition of stroke symptoms, risk/protective factors, and stroke response in Vietnamese communities living in SWS and investigate whether culturally tailored education sessions could improve stroke literacy.

**Methods:**

A prospective interventional study evaluated a single 1.5-h stroke education workshop. Data included pre/post-education surveys, participant demographics and stroke literacy. Change in literacy and contributing factors were analysed.

**Results:**

There were 195 participants in three sessions. Stroke symptoms were recognised by the majority [Face:(56.4 %), Arms:(66.7 %), Speech:(61.5 %)], with 52.8 % identifying all three whilst 29.2 % recognised none. Most participants were confident calling an ambulance (60.0 %), aware of diabetes as a risk factor (73.9 %) and recognised healthy diet/exercise (82.5 %) as protective factors. Post-education, 24.6 % recognised more symptoms, with 73.4 % identifying all three and only 16.6 % recognising none. 33.1 % were more confident calling an ambulance, 32.4 % more aware of diabetes mellitus, and 29.8 % more aware of diet/exercise. Smaller group size [OR = 2.83, 95 %CI = 1.15–6.96 (*p* = 0.024), lower age [OR = 0.93, 95 %CI = 0.87–1.00 (*p* = 0.037)] and lower baseline literacy [OR = 6.38, 95 %CI = 2.48–16.41 (*p* < 0.001)] were significantly associated with improved stroke literacy post-education.

**Conclusion:**

Stroke literacy in the SWS Vietnamese community improved with a single culturally tailored education session. This study underscores the importance of tailored educational interventions and highlights the need for strategies addressing low baseline literacy and age-related barriers.

## 1 Introduction

Culturally and linguistically diverse (CALD) communities have a higher rate of stroke and worse post-stroke clinical outcomes than the general population [1,2]. Factors contributing to these health disparities include difficulty navigating an unfamiliar health system, socioeconomic and language barriers, as well as the potential for discrimination and racism [3,4]. CALD communities often have lower health literacy, finding more difficulty accessing and understanding health information to seek appropriate services in a timely manner to maintain good health and wellbeing [3]. Lower stroke health literacy may be an important contributing factor towards worse stroke outcomes. Stroke health literacy includes the recognition of stroke symptoms, risk/protective factors, and stroke response in terms of knowledge of urgency and confidence calling an ambulance [5,6].

Within CALD communities, the Vietnamese population represents a significant subgroup that warrants focused investigation due to unique risk factors and barriers to care. In Australia, the Vietnamese population is continuously growing, increasing by 32.8 % from 2012 to 2022 to become the sixth largest migrant community and 1.1 % of the country's population [7]. Australia is home to the second largest Vietnamese community after the United States (US) within developed countries [8]. In the 2016 census, 84,130 people born in Vietnam were living in New South Wales (∼1.1 % of state population) [9,10]. A substantial proportion of the Vietnamese population resides in South-Western Sydney (SWS), where 10.8 % of SWS residents are of Vietnamese ancestry and Vietnamese is the most commonly spoken language at home (11.4 % of households) [11].

In Vietnam, stroke is the leading cause of death and disability [12]. Vietnam-born Australians are also more susceptible to stroke, having higher prevalences of risk factors including diabetes, reduced physical activity and lower levels of vegetable consumption compared to the Australian-born population [8,13]. A recent study in SWS Vietnamese communities reported significantly higher rates of stroke risk factors of hypertension and dyslipidaemia, as well as stroke aetiologies of intracranial atherosclerotic disease and intracerebral haemorrhage compared to Caucasian patients [14]. This could be attributed to environmental factors, including poorer diets with higher sodium, fats and processed foods, psychological stress, and acculturation, as significant lifestyle changes occur during assimilation into a new cultural environment [15]. These findings highlight the need for targeted stroke education for migrant Vietnamese-Australians.

Stroke health literacy data in Vietnamese communities is limited internationally. The current literature on Vietnamese Americans shows a lack of stroke literacy and cost-effective education strategies. Nguyen et al. reported that only 22 % of Vietnamese Americans (*n* = 4254) could identify five correct symptoms of stroke and less than 5 % knew the correct symptoms and action to call 911 [5]. Ton et al. supported this finding, reporting that Vietnamese immigrants to the US did not mention dysphasia, blurred vision, weakness, or sudden confusion at all when asked about stroke symptoms [6]. A 2022 study in the US Hmong population, an ethnic group in Vietnam, found that most of the respondents did not know any stroke risk factors [16]. There is a lack of stroke health literacy data in Vietnamese communities living in Australia.

Culturally sensitive practices in healthcare are increasingly necessary to close the gap in health outcome disparities in the CALD community. Improving symptom recognition, reducing delayed presentations to hospital and managing stroke risk factors are critical. In the Vietnamese community, where stroke is highly prevalent, primary preventative measures that are culturally tailored are of paramount importance to overcome the barriers that deter appropriate healthcare from being sought. Educational interventions in stroke and other diseases are known to be effective in enhancing health literacy among CALD communities [17]. Community-based studies have highlighted the benefits of bilingual and bicultural education in CALD communities [18,19].

There are limited studies that research the effect of an educational intervention on stroke literacy in Vietnamese communities. This knowledge gap highlights the need for further studies on educational interventions in Vietnamese populations, who face significant health disparities, especially for stroke. This study aimed to characterise the current level of stroke literacy in SWS Vietnamese communities and investigate whether culturally specific evidence-based education interventions can be designed and delivered to improve stroke literacy.

## 2 Materials and methods

This was a prospective interventional study analysing the effectiveness of educational interventions that aimed to improve stroke literacy in SWS Vietnamese communities. Vietnamese community members who attended the Vietnamese New Year (Tet) Festival were invited to complete a stroke literacy survey available in both English (Appendix A1) and Vietnamese (Appendix A2) at a Stroke and Vascular Health stall to assess the baseline stroke literacy level of the community. The results of this festival survey (Appendix A3) were used to design a 1.5 h culturally tailored stroke education workshop and pre- and post-education survey. This was delivered by clinicians from the SWS Local Health District (SWSLHD) Sydney Brain Centre in collaboration with members from Multicultural Services and the University of New South Wales (UNSW), in three separate education sessions for the Vietnamese community. A certified Vietnamese interpreter was present to assist staff with the delivery of the stroke education session and survey completion. The first and second sessions were delivered in a senior citizen centre with 6 facilitators and 1 interpreter, while the third was delivered in a community club setting with 6 facilitators and 2 interpreters. The facilitators consisted of members of the research team and co-authorship, three of whom were Vietnamese speaking. The education was delivered in Vietnamese and involved an interactive 15-min stroke case study, then a 20-min lecture on stroke symptoms, risk factors, prevention strategies and what to do in the event of a stroke and how to call an ambulance. Clinicians then facilitated a 45-min question-and-answer session. Participants completed a stroke literacy survey translated into Vietnamese (Appendix A2) before and after a stroke education session to assess baseline literacy and whether their stroke literacy had improved after a single education session. Participants were offered access to FAS (Face, Arms, Speech) symptoms leaflets translated into Vietnamese and Stroke Foundation – Australia, (https://strokefoundation.org.au/) reading material after each education session.

Ethics approval (2019/STE14038) for this study was granted by the SWSLHD Human Research Ethics Committee.

Participants were eligible for the study if they were born in Vietnam or of Vietnamese ancestry, over 18 years old, and were able to engage with study materials. The data collected from all three education sessions were pooled. Demographic data collected included age, gender, country of birth and years lived in Australia. Participants also reported whether they had experienced a stroke themselves or in a family member, and level of health literacy indicated by the frequency of help needed when filling out health-related documents.

The distribution of continuous data was evaluated prior to analyses using the Shapiro-Wilk test, with parametric data represented as mean and standard deviation (SD), and non-parametric data represented as median and interquartile range (IQR).

In this study, health literacy was measured by the frequency of help needed to fill in health-related documents, which was reported on a 5-point Likert scale ranging from “never” to “all the time” [20,21]. To allow for meaningful analysis, the results were dichotomised into high (scores 3–5) and low (scores 1–2) health literacy If participants circled two responses, the higher value was chosen.

Stroke literacy was defined in this study as recognising FAS symptoms confidence in calling an ambulance, awareness of diabetes as a risk factor and awareness of healthy diet/exercise as protective factors. Descriptive analyses were conducted to compare results from the pre- and post-education survey for each indicator of stroke literacy. The number and percentages of participant responses for each question were calculated for pre- and post-education surveys. Four logistic regression models were completed to explore factors associated with change in each component of stroke literacy we assessed. Statistical analysis was conducted using IBM SPSS Statistics for Windows, version 26 (IBM Corp Armonk, NY: USA).

## 3 Results

### 3.1 Participant characteristics

The sample population consisted of 195 participants, of which 33 (19.9 %) were male, 133 (80.1 %) female and 29 (14.9 %) did not report their gender (Table 1). Of 176 participants who supplied their age, the median age was 69 (IQR 64, 74). Of 173 participants who answered, 167 (96.5 %) were born in Vietnam and 6 (3.5 %) outside of Vietnam. Participants had resided in Australia for a median time of 31 years (IQR 19, 40). Of 175 participants who answered, 56 (68.0 %) reported a stroke in themselves or a family member. There was an even distribution between participants with high health literacy (51.5 %) and low health literacy (48.5 %). For details on the raw health literacy data, refer to Appendix B. There were three education sessions, with 33 (16.9 %) participant responses collected in the first session, 52 (26.7 %) in the second and 110 (56.4 %) in the third.

**Table 1:** Demographics of sample (*n* = 195).

Characteristic	Description	Results
Age (*n* = 176), mean (SD)		69.3 (7.58)
Sex (*n* = 166), n (%)	Male	33 (19.9 %)
	Female	133 (80.1 %)
Country of Birth (*n* = 173), n (%)	Vietnam	167 (96.5%)
	Cambodia	3 (1.7 %)
	Laos	1 (0.6 %)
	Australia	2 (1.2 %)
Years lived in Australia (*n* = 163), median (IQR 25, 75)		31 (19, 40)
Have had a stroke or family with stroke (*n* = 175), n (%)	Yes	56 (32.0 %)
	No	119 (68.0 %)
Health literacy (*n* = 167), n (%)	Low	81 (48.5%)
	High	86 (51.5 %)
Education session attendance, n (%)	1	33 (16.9%)
	2	52 (26.7 %)
	3	110 (56.4 %)

### 3.2 Pre- and post-education changes in stroke literacy

Just over half of the participants (52.8 %) could recognise all three FAS stroke symptoms at baseline, and less than a third (29.2 %) recognised no symptoms (Table 2). After the education session, 73.4 % could recognise all three symptoms, while 16.6 % were still unable to recognise any. Individually, there was higher baseline awareness of ‘difficulty moving arms’ (66.7 %), followed by ‘slurred speech or confused’ (61.5 %), then ‘drooping face’ (56.4 %).

**Table 2:** Comparison between pre- and post-education survey results on stroke literacy among the Vietnamese population in SWS.

Characteristic	Pre-education n (%)	Post-education n (%)
**Recognition of Stroke Symptoms (FAS)**	n = 195	*n* = 169
F - Drooping face	110 (56.4 %)	131 (67.2 %)
A - Difficulty moving arms	130 (66.7 %)	135 (69.2 %)
S - Slurred speech or confused	120 (61.5%)	131 (67.2 %)
Number of FAS symptoms identified		
0	57 (29.2 %)	28 (16.6 %)
1	19 (9.7 %)	9 (5.3 %)
2	16 (8.2 %)	8 (4.7 %)
3	103 (52.8 %)	124 (73.4 %)
**Confidence in Calling an Ambulance**	*n* = 185	*n* = 165
Not at all confident, not confident or unsure	74 (40.0 %)	28 (16.9%)
Confident or very confident	111 (60.0 %)	137 (83.1 %)
**Awareness of Diabetes as a Stroke Risk Factor**	*n* = 180	n = 163
Strongly disagree, disagree or unsure	47 (26.1 %)	16 (9.8%)
Agree or strongly agree	133 (73.9%)	147 (90.2 %)
**Awareness of Healthy Diet and Exercise as Stroke Protective Factors**	*n* = 183	n = 165
Strongly disagree, disagree or unsure	32 (17.5%)	8 (4.8%)
Agree or strongly agree	151 (82.5 %)	157 (95.2 %)

Confidence in calling an ambulance increased from 60 % to 83.1 % after the education sessions, and there was a reduction in the proportion of participants who were unsure about calling an ambulance (24.3 % to 9.7 %) or who were not confident (15.7 % to 7.2 %). There was a high awareness of diabetes as a stroke risk factor (73.9 %) and awareness of healthy diet/exercise as protective factors for stroke (82.5 %). For more details on pre-collapsed data for confidence calling an ambulance and awareness of stroke risk/preventative factors, refer to Appendix C.

Overall, the education sessions saw a 20.6 % increase in participants who could recognise all three FAS symptoms, 23.1 % increase in participants who were confident or very confident calling an ambulance, 16.3 % and 12.7 % increase in participants who respectively were aware of diabetes, and healthy diet and exercise as stroke risk/protective factors (Table 2).

A quarter to a third (24.6–33.1 %) of participants reported improvement for all four indicators of stroke literacy (Table 3). Approximately half (42.3–57.8 %) of participants with high baseline literacy and 3.9–15.3 % with low baseline literacy showed no change after the education sessions. A small portion of participants reported reduced knowledge (Appendix D).

**Table 3:** Change in stroke literacy after education.

	Symptom recognition and confidence calling an ambulance		Awareness of stroke risk/protective factors	
Change in stroke literacy by baseline	FAS (*n* = 163)	Ambulance (*n* = 163)	Diabetes (*n* = 154)	Healthy diet and exercise (*n* = 161)
Improved, n (%)	40 (24.6 %)	54 (33.1 %)	50 (32.4 %)	48 (29.8 %)
No improvement, n (%)	123 (65.9 %)	109 (66.8 %)	104 (67.6 %)	113 (73.9 %)

Abbreviations: FAS = Face, Arms, Speech.

### 3.3 Factors associated with stroke literacy improvement

In unadjusted analyses, there was a significant difference in the change in FAS recognition (*p* = 0.014), confidence in calling an ambulance (*p* = 0.001) (Table 4a), awareness of diabetes (*p* = 0.012) and healthy diet/exercise (*p* = 0.046) as risk/protective factors between sessions 1, 2 and 3 (Table 4b). There was also a significant correlation between level of health literacy and change in FAS symptom recognition (*p* = 0.007), where participants with high baseline stroke literacy (16.7 %) showed less improvement than those with a low level of health literacy (36.2 %) (Table 4a).

**Table 4a:** Change in stroke literacy (FAS symptom recognition and confidence calling an ambulance) after education with regards to differences within participants.

	FAS symptoms recognition				Confidence calling ambulance			
	No improvement, n (%)		Improved, n (%)	*p*-value	No improvement, n (%)		Improved, n (%)	p-value
Session	n = 163				n = 163			
1		16 (55.2 %)	13 (44.8 %)			13 (40.6 %)	19 (59.4 %)	
2		30 (75.0 %)	10 (25.0 %)			32 (80.0 %)	8 (20.0 %)	
3		77 (81.9%)	17 (18.1 %)	0.014		64 (70.3 %)	27 (29.7 %)	0.001
**Sex**	*n* = 143				*n* = 144			
Male		22 (78.6 %)	6 (21.4 %)			21 (72.4 %)	8 (27.6 %)	
Female		90 (78.3 %)	25 (21.7 %)	0.971		78 (67.8%)	37 (32.2 %)	0.634
**Stroke experience**	*n* = 149				n = 154			
Yes		42 (80.8%)	10 (19.2 %)			35 (66.0 %)	18 (34.0 %)	
No		69 (71.1 %)	28 (28.9%)	0.198		68 (67.3 %)	33 (32.7 %)	0.872
**Level of health literacy**	*n* = 147				*n* = 148			
High		65 (83.3 %)	13 (16.7 %)			56 (70.9%)	23 (29.1 %)	
Low		44 (63.8 %)	25 (36.2 %)	0.007		6 (62.3 %)	26 (37.7 %)	0.269
**Continuous variables, mean (SD)**								
**Age**	*n* = 152				*n* = 153			
		68.91 (7.41)	67.97 (7.05)	0.502		69.30 (7.67)	68.25 (6.65)	0.407
**Number of years lived in Australia**	*n* = 142				n = 142			
		28.99 (12.22)	28.58 (13.02)	0.867		29.95 (12.07)	27.83 (13.14)	0.341

Abbreviations: FAS = Face, Arms, Speech.

**Table 4b:** Change in stroke literacy (awareness of diabetes as a stroke risk factor and healthy diet/exercise as preventative factors) after education with regards to differences within participants.

	Awareness of diabetes as a stroke risk factor				Awareness of healthy diet/exercise as stroke preventative factors			
	No improvement, n (%)		Improved, n (%)	p-value	No improvement, n (%)		Improved, n (%)	p-value
**Session attended**	n = 154				n = 161			
1		14 (50.0 %)	14 (50.0 %)			24 (85.7 %)	4 (14.3 %)	
2		32 (84.2 %)	6 (15.8%)			29 (76.3 %)	9 (23.7 %)	
3		58 (65.9%)	30 (34.1 %)	0.012		60 (63.2 %)	35 (36.8 %)	0.046
**Sex**	*n* = 136				n = 136			
Male		24 (82.8%)	5 (17.2 %)			17 (60.7 %)	11 (39.3 %)	
Female		71 (66.4 %)	36 (33.6 %)	0.088		77 (71.3 %)	31 (28.7 %)	0.280
**Stroke experience**	n = 144				*n* = 146			
Yes		63 (67.7 %)	30 (32.3 %)			32 (71.1 %)	13 (28.9%)	
No		35 (68.6 %)	16 (31.4 %)	0.913		71 (70.3 %)	30 (29.7 %)	0.912
**Level of health literacy**	*n* = 141				*n* = 140			
High		49 (63.6 %)	28 (36.4 %)			50 (69.4 %)	22 (30.6 %)	
Low		45 (70.3 %)	19 (29.7 %)	0.402		47 (69.1 %)	21 (30.9%)	0.967
**Continuous variables, Mean (SD)**								
**Age**	n = 143				*n* = 145			
		69.54 (7.01)	68.59 (8.13)	0.474		69.84 (7.48)	68.80 (8.32)	0.456
**Number of years lived in Australia**	*n* = 133				*n* = 135			
		28.93 (12.49)	30.98 (11.32)	0.361		28.05 (12.02)	31.79 (12.67)	0.101

Logistic regression revealed that age and session attended were only statistically significant for change in FAS recognition (Table 5). With every 1-year increase in age, participants were 7 % less likely to show a change in FAS knowledge after the education session [OR = 0.93, 95 %CI = 0.87–1.00 (*p* = 0.037)]. Participants who attended sessions 1 or 2 were 3 times more likely to improve in FAS recognition compared to session 3 [OR = 2.83, 95 %CI = 1.15–6.96 (*p* = 0.024)] (Table 5). Participants who knew no FAS symptoms at baseline were significantly more likely than those who knew one or more FAS symptoms to improve after education [OR = 6.38, 95 %CI = 2.48–16.41 (*p* < 0.001)].

**Table 5:** Logistic regression model of predictors of stroke literacy improvement after education.

Independent Variable	OR (95 % CI)	p-value
**FAS recognition**		
Increasing age	0.93 (0.87–1.00)	0.037
Attended session 1 or 2	2.83 (1.15–6.96)	0.024
Female	0.75 (0.24–2.38)	0.628
Knew no FAS symptoms at baseline	6.38 (2.48–16.41)	<0.001
**Confidence calling an ambulance**		
Increasing age	0.95 (0.89–1.00)	0.079
Attended session 1 or 2	0.81 (0.36–1.85)	0.617
Female	0.77 (0.27–2.21)	0.625
Lower confidence	8.13 (3.51–18.84)	<0.001
**Awareness of diabetes as a stroke risk factor**		
Increasing age	0.99 (0.93–1.06)	0.821
Attended session 1 or 2	1.35 (0.55–3.32)	0.514
Female	0.35 (0.10–1.24)	0.103
Lower awareness	12.67 (4.59–35.00)	<0.001
**Awareness of healthy diet and exercise as stroke protective factors**		
Increasing age	0.98 (0.93–1.03)	0.475
Attended session 1 or 2	1.86 (0.82–4.23)	0.137
Female	1.78 (0.71–4.48)	0.220
Lower awareness	1.00 (0.34–2.96)	0.998

Abbreviations: FAS = Face, Arms, Speech.

Participants with lower baseline confidence calling an ambulance were significantly more likely to improve their confidence after the session [OR = 8.13, 95 %CI = 3.51–18.84 (p < 0.001)], and participants with lower baseline awareness of diabetes were also significantly more likely to improve their awareness after the education session [OR = 12.67, 95 %CI = 4.59–35.00, (p < 0.001)]. No variables were significantly associated with change in awareness of diet/exercise for stroke prevention (Table 5).

### 3.4 Insights from question-and-answer sessions

Questions asked during the question-and-answer session included uncertainty about what to specifically say when communicating with an emergency services operator after calling an ambulance if they don't speak or lacked confidence communicating in English. The phrase “Stroke-FAST-Vietnamese” was well-received as a potential phrase to alert the operator and link the caller to a Vietnamese interpreter. The word ‘FAST’ was perceived by participants as an indicator of the importance of time and severity of the emergency rather than an acronym for stroke symptoms. Participants in all three sessions asked about the best physical position for a person experiencing acute stroke while waiting for the ambulance, and what to do if they are living alone, can't speak due to dysarthria and dysphasia, or can't access a phone due to stroke severity. The potential benefits of a vital alert system and how to access these were also discussed.

## 4 Discussion

The present study found that a single education session could improve short-term stroke literacy in a Vietnamese community living in Australia. The findings of this study were comparable to other community-based educational intervention studies. The 20.6 % (52.8 % to 73.4 %) increase in participants who could recognise all three FAS symptoms after the education session was similar to a US study which reported a 23 % (42 % to 65 %) improvement in recognition of all five stroke symptoms after a 4-month educational intervention in older adults. However, the population of this US study differed from ours as it did not have a CALD focus [19]. Nishikawa et al. also found that 15.6 % of participants improved from selecting less than 5 correct stroke symptoms to selecting all 5 correct stroke symptoms after a two-year intervention in Japan [22]. Zhong et al. reported an 11.8 % increase (from 7.1 % to 18.9 %) in participant recognition of the FAST acronym after a one-year educational intervention in China [23]. These studies show no clear correlation between intervention duration and increase in knowledge, and so these different results may be attributed to varying cultural differences and baseline knowledge levels of participants, as well as differences in the delivery of the education intervention itself.

The present study reported lower change in awareness of stroke risk factors. A US study reported a 157 % increase in diabetes knowledge scores using the Spoken Knowledge of Diabetes in Low Literacy Patients with Diabetes (SKILLD) scale in 186 Hispanic patients with low diabetes literacy after weekly education sessions administered over 8 weeks [18]. The present study showed more modest improvement in diabetes awareness (32.4 % improvement) and stroke symptom recognition than previous research [18,23]. In a population with similar baseline knowledge level to our study, the variation in improvement may be accounted for by the differences in modes of delivery of the educational interventions. The previous studies included the distribution of leaflets, booklets and recorded lectures over a longer period as opposed to this study, which only delivered a single 1.5-h education session.

Participants with lower baseline stroke literacy were significantly more likely to demonstrate improved stroke literacy after the education session. This showcases that less-informed individuals benefit more from culturally specific education interventions. In contrast, approximately half of the participants reported high baseline stroke literacy and many participants had personal experience or a family member affected with stroke, therefore had limited scope to improve.

With every increase of one year in age, participants were 7 % less likely to show a change in FAS knowledge after the education session. This may be due to cognitive decline as part of ageing, including reduced memory recall and speed of processing information [24]. These findings highlight that more prolonged and repetitive education and/or the content of educational strategies should be tailored to the needs of older participants. Although the present study comprised a high proportion of female participants, gender was not significantly associated with improving stroke literacy, suggesting that this education strategy has broad applicability irrespective of gender.

Participants who attended sessions 1 or 2 were three times more likely to improve FAS recognition than session 3. As the baseline stroke literacy of participants in session 3 did not notably differ from those in other sessions (Appendix E), this result may relate to the large number of attendees to session 3 which had the smallest ratio of facilitators to participants. This finding is supported by Davis & Davis, who reported that large group education sessions may produce little to no change in performance, although can still be effective with increased interactivity, such as including a question-and-answer session [25]. Higher facilitator to participant ratios allowed for more questions from the audience to be addressed in a limited time frame and more assistance for participants who had difficulty completing the surveys. Participants may have also felt more engaged with the materials and more comfortable asking questions in smaller groups. The setting may also have influenced the education as Session 3 delivered in a large conference venue in a club setting, compared to smaller community-based centres (sessions 1 and 2). In session 3 audibility was potentially an issue as facilitators had to walk back and forth with a microphone between participants and facilitators. This may have disrupted the flow and taken time off the question-and-answer segment, which was less of an issue in sessions 1 and 2 as they were delivered in a classroom or hall where microphones were unnecessary.

This study is the largest prospective interventional stroke study on Vietnamese communities in Australia to date. The team consisted of bilingual and clinical staff with skillsets spanning various areas of expertise, collaborating to provide accurate and culturally tailored education to the Vietnamese community. A limitation of the study is that it primarily assesses immediate outcomes without providing insights into long-term retention of knowledge or changes in health behaviours. The post-education survey was conducted immediately after the education session, and so recency bias may have shown a higher level of improvement than actuality. Prospective studies that follow participants after a few months and/or at one year will provide a better indicator of long-term retention and application of knowledge. The transferability or generalisability of the findings is also unclear. Similar studies involving Vietnamese communities in other health districts may be of value. Another limitation of the study includes the survey design for FAS symptom recognition, which required participants to tick any symptoms recognised instead of a ‘yes/no’ response for each symptom or a free-text response. As a result, the study assumed that unticked responses represented a lack of recognition, which may not necessarily be the case. Participants may have left post-survey responses unticked due to fatigue or lack of interest. Furthermore, listing out the FAS symptoms in order may have prompted some participants to tick every box. Having a free-answer question or re-phrasing the question with a ‘yes/no’ answer may have provided a better measure of true symptom recognition. Alternatively, including decoy answers like Nishikawa et al. when asking participants to tick the stroke symptoms could better differentiate whether the participants were only selecting all answers or have a true understanding of stroke symptoms [22]. Another limitation of the survey design was that it did not ask whether participants had attended a previous education session, which would have influenced their baseline literacy.

Feedback from the question-and-answer session also highlighted how the FAST acronym does not translate well nor retain its meaning in Vietnamese. “Stroke-FAST-Vietnamese”, which was positively received, has the potential to be used in further education sessions in the Vietnamese community and disseminated to other CALD communities. Stroke awareness campaigns such as FAST are designed for populations with a level of English proficiency resulting in some countries considering the implementation of different strategies such as Stroke 1–2-0 in China [26]. The letters within the FAST acronym do not necessarily translate in a meaningful way in other languages, for example, the Vietnamese words for ‘fast’ and ‘time’ are ‘nhanh’ and ‘thời gian’, respectively. Similarly, for migrants with low English proficiency living in a predominantly English-speaking country, stroke awareness strategies that are not tailored to the relevant ethnic population or demonstrate cultural sensitivity may be less effective. Future research could be conducted to validate the acceptability of phrases such as “Stroke-FAST-Vietnamese”, or “Stroke-FAST-Language” for both consumers and emergency services.

There are other types of community-based participatory research approaches which have been reported to be effective in addressing CALD stroke health needs including multi-media (internet) based health interventions [27], school class-based stroke knowledge and behaviour modification programs [28] and community stakeholder intervention programs such as the US urban beauty shop stroke education project [29]. A co-design approach with consumers with lived experience of stroke could be incorporated in future studies, to develop a stronger evidence-based program that is culturally specific to the Vietnamese community. Future directions include the creation of a sustainable program from the current education materials, which could involve recorded videos of presentations delivered by health professionals to showcase at future events, expanding the facilitator pool by training more staff to deliver the same presentations and creating an online forum where community members can ask questions, allowing more time flexibility for staff to address them. Including answers to the commonly asked questions from the question-and-answer session would be beneficial when developing future education materials. Future studies could be conducted to investigate the effectiveness of this education strategy delivered in other languages and tailored to other cultures in the CALD community.

## 5 Conclusion

This study found mixed levels of stroke literacy in a SWS Australia Vietnamese community, which could be effectively improved by a single education session. The results and observations inform the development of the current education materials to include what the community was most receptive to, whilst improving on the sections where participants showed the least improvement. Evaluation of the outcomes from this study is valuable as its application can create more effective and sustainable education for delivery to Vietnamese and other CALD communities that face similar disparities in stroke outcomes.

## CRediT authorship contribution statement

**Jessica Ly:** Writing – original draft, Resources, Methodology, Investigation, Formal analysis, Data curation. **Christopher Blair:** Supervision, Resources, Project administration, Methodology, Investigation, Formal analysis, Conceptualization. **Helen Badge:** Writing – original draft, Resources, Methodology, Investigation, Formal analysis. **Michael Camit:** Resources, Methodology, Investigation. **Khoi Do:** Resources, Methodology, Investigation. **Timmy Pham:** Resources, Methodology, Investigation. **Nicola Chappelow:** Resources, Methodology, Investigation. **Dennis J. Cordato:** Supervision, Resources, Project administration, Methodology, Investigation, Formal analysis, Conceptualization. **Mark W. Parsons:** Supervision, Resources, Project administration, Methodology, Investigation, Formal analysis, Conceptualization.

## Declaration of competing interest

Dennis Cordato reports financial support was provided by Sydney Partnership for Health Education Research and Enterprise. If there are other authors, they declare that they have no known competing financial interests or personal relationships that could have appeared to influence the work reported in this paper.

## Figures and Tables

**Table:**
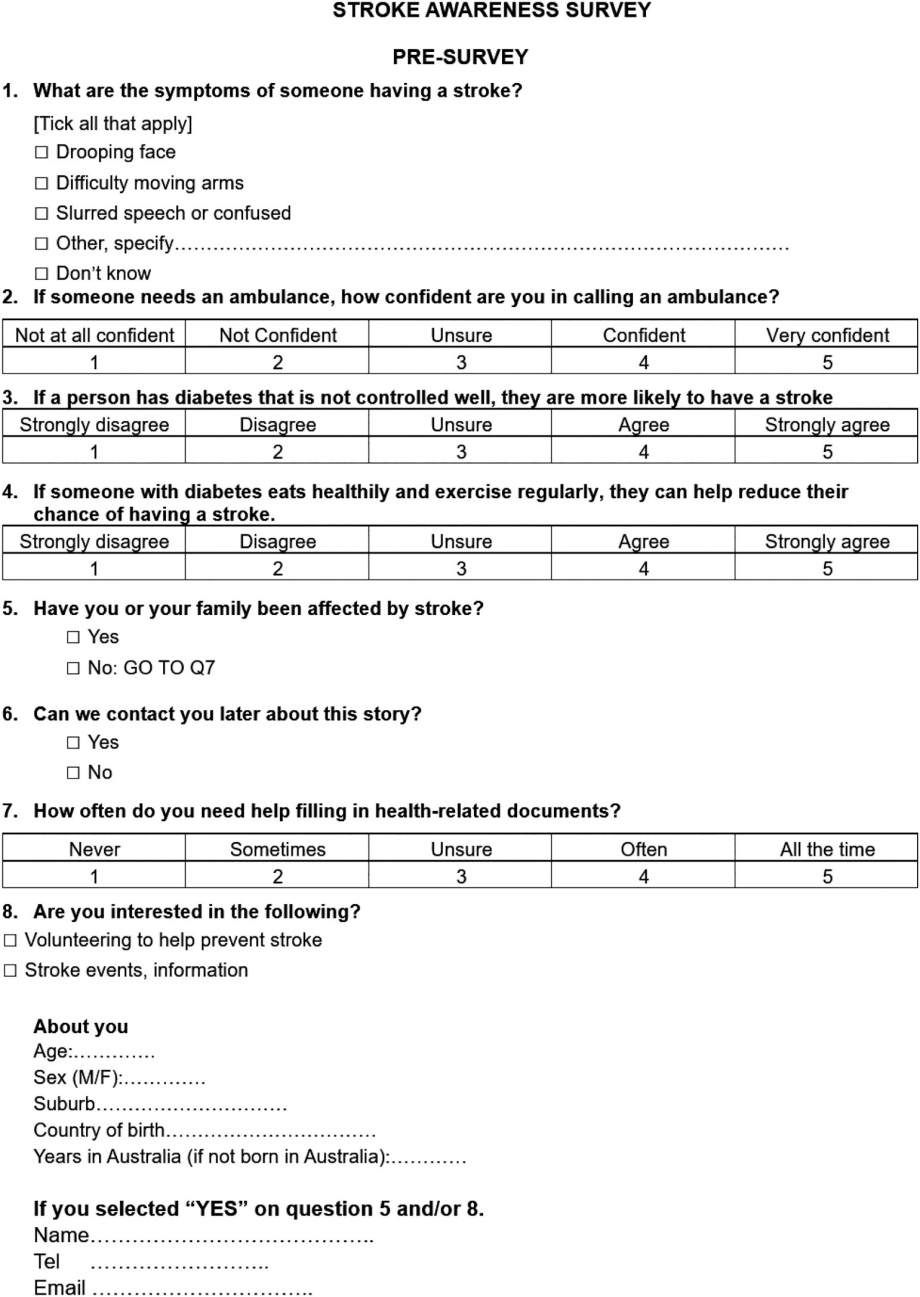

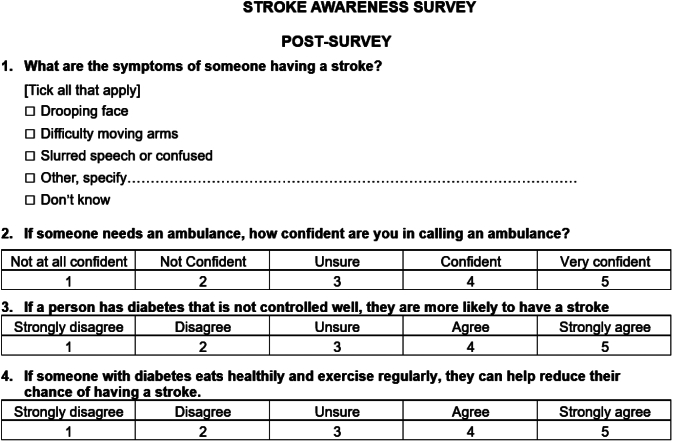


**Table:**
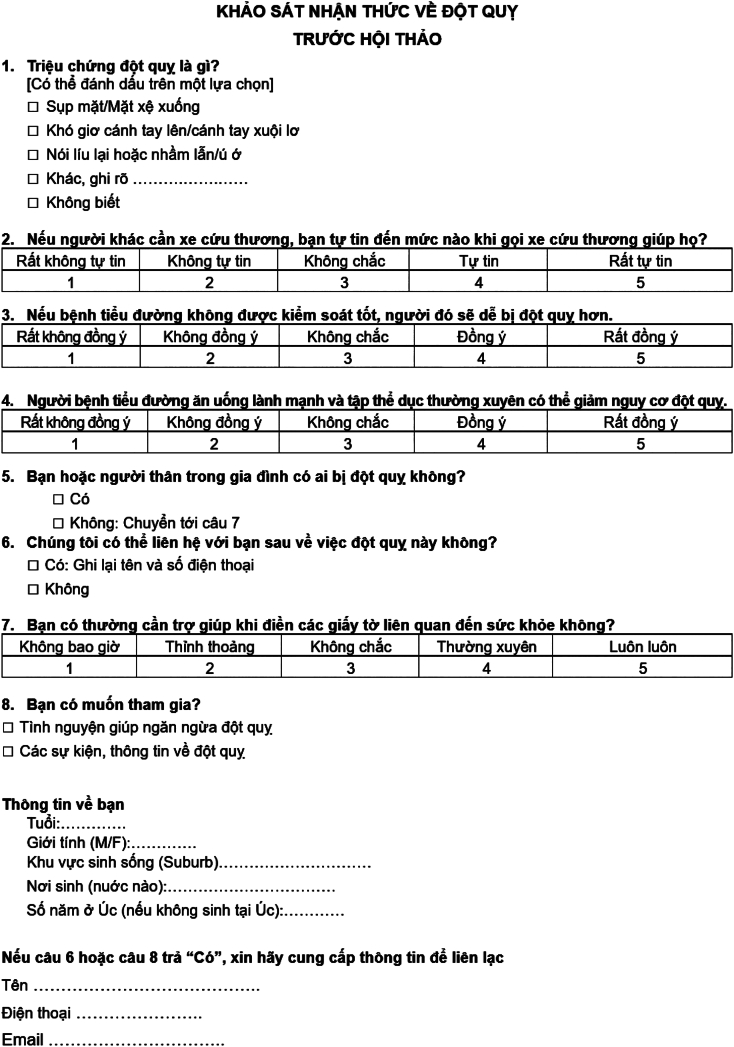

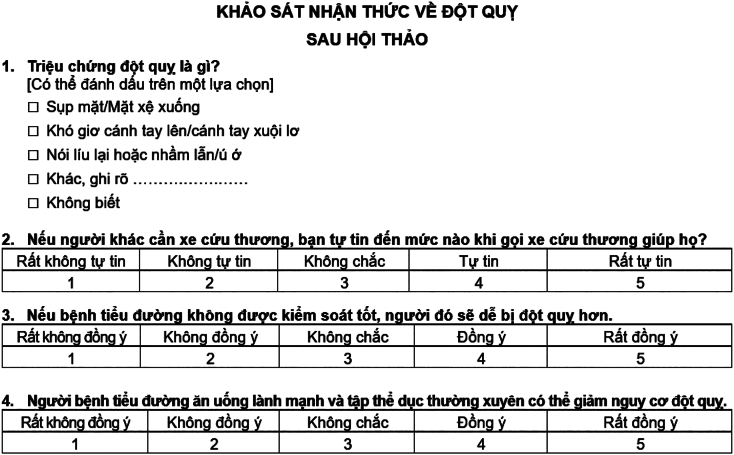


**Table:** Raw data for frequency of help required when filling in health-related documents (*N* = 167).

Characteristic	Description	Results, n (%)
Frequency of help required when filling in health-related documents	Never	17 (10.2 %)
	Sometimes	69 (41.3 %)
	Unsure	10 (6.0 %)
	Often	41 (24.6 %)
	All the time	30 (18.0 %)

**Table:** Raw data for the comparison between pre- and post-education survey results for confidence calling an ambulance and awareness of stroke risk/protective factors.

Characteristic	Pre-education, n (%)	Post-education, n (%)
**Confidence in calling an ambulance**	n = 185	n = 165
Not at all confident	15 (8.1 %)	7 (4.2 %)
Not confident	14 (7.6 %)	5 (3.0 %)
Unsure	45 (24.3 %)	16 (9.7 %)
Confident	60 (32.4 %)	76 (46.1 %)
Very confident	51 (27.6 %)	61 (37.0 %)
**Awareness of diabetes as a stroke risk factor**	n = 180	n = 163
Strongly agree	11 (6.1 %)	9 (5.5%)
Disagree	5 (2.8%)	2 (1.2 %)
Unsure	31 (17.2 %)	5 (3.1 %)
Agree	76 (42.2 %)	77 (47.2 %)
Strongly agree	57 (31.7 %)	70 (42.9%)
**Understanding of the link between healthy diet and exercise as preventative factors for stroke**	n = 183	n = 165
Strongly disagree	12 (6.6 %)	4 (2.4 %)
Disagree	0 (0.0 %)	0 (0.0 %)
Unsure	20 (10.9%)	4 (2.4 %)
Agree	81 (44.3 %)	73 (44.2 %)
Strongly agree	70 (38.3 %)	84 (50.9 %)

**Table:** Pre-collapsed change in stroke awareness after education session by baseline awareness.

	Stroke questions		Awareness of stroke risk/preventative factors	
Change in confidence/awareness by baseline	FAS (N = 163)	Ambulance (N = 163)	Diabetes (N = 154)	Healthy diet and exercise (N = 161)
Improved, N (%)	40 (24.6 %)	54 (33.1 %)	50 (32.4 %)	48 (29.8 %)
No change	118 (62.8 %)	94 (57.6 %)	82 (53.3 %)	97 (64 %)
Subgroup according to baseline awareness/confidence				
High, n (%)	95 (48.7 %)	69 (42.3 %)	76 (49.4 %)	93 (57.8 %)
Low, n (%)	23 (14.1 %)	25 (15.3 %)	6 (3.9 %)	4 (6.2 %)
Reduced, n (%)	5 (3.1 %)	15 (9.2 %)	22 (14.3 %)	16 (9.9 %)

**Table:** Pre-education survey results according to session.

Characteristic	Session 1, n (%)	Session 2, n (%)	Session 3, n (%)
**Recognition of Stroke Symptoms (FAS)**			
Number of FAS symptoms identified	*n* = 33	*n* = 52	*n* = 110
0	12 (36.4 %)	13 (25.0 %)	32 (29.1 %)
1	6 (18.2 %)	5 (9.6 %)	8 (7.3 %)
2	2 (6.1 %)	3 (5.8 %)	11 (10.0 %)
3	13 (39.4 %)	31 (59.6 %)	59 (53.6 %)
**Other Knowledge**			
Confidence in calling an ambulance	n = 33	*n* = 49	*n* = 103
Not at all confident, not confident or unsure	14 (42.4 %)	22 (44.9 %)	38 (36.9 %)
Confident or very confident	19 (57.6 %)	27 (55.1 %)	65 (63.1 %)
Awareness of diabetes as a stroke risk factor	*n* = 30	*n* = 49	*n* = 101
Strongly disagree, disagree or unsure	6 (20.0 %)	8 (16.3 %)	33 (32.7 %)
Agree or strongly agree	24 (80.0 %)	41 (83.7 %)	68 (67.3 %)
Awareness of healthy diet and exercise as preventative factors for stroke	*n* = 32	*n* = 48	n = 103
Strongly disagree, disagree or unsure	6 (18.8%)	5 (10.4 %)	21 (20.4 %)
Agree or strongly agree	26 (81.3 %)	43 (89.6 %)	82 (79.6 %)

## Appendix A Appendix

### A.3 Tet festival preliminary data

A descriptive study was performed by conducting surveys on community members at the 2024 Fairfield Showground Tet Festival. Survey questions included whether participants had personal experience with stroke, confidence in calling an ambulance and filling out health-related documents, and recognition of Face, Arm, Speech, Time (FAST) signs. Of the 183 participants, 44.9 % reported that they or their family members have been affected by stroke. Less than 50 % of the participants were aware of FAST signs, with 43.7 % able to identify stroke signs related to the face, 46.4 % the arm, and 44.8 % speech. More participants felt confident calling an ambulance than not (37 % very confident and 41 % confident versus 3 % not confident, 4 % not at all confident). Such findings inform the development of health interventions culturally tailored to the Vietnamese community and their level of stroke literacy, which may ultimately improve stroke prevention and outcomes.
